# CAREGIVERS’ POOR SLEEP AND PLASMA BIOMARKERS FOR NEURODEGENERATION

**DOI:** 10.1093/geroni/igae098.3373

**Published:** 2024-12-31

**Authors:** Yeonsu Song, Sarah Choi, Raeanne Moore, Alexander Erickson, Karen Camacho, Monica Cappelletti, Cathy Alessi, Jennifer Martin

**Affiliations:** University of California Los Angeles, Los Angeles, California, United States; University of California Los Angeles, Los Angeles, California, United States; University of California San Diego, La Jolla, California, United States; VA Greater Los Angeles Healthcare System, Los Angeles, California, United States; Greater Los Angeles VA Health Care System, North Hills, California, United States; University of California Los Angeles, Los Angeles, California, United States; VA Greater Los Angeles Healthcare System, Los Angeles, California, United States; VA Greater Los Angeles Healthcare System, Los Angeles, California, United States

## Abstract

Family caregivers of people living with dementia (PLWD) have an increased risk of dementia. Poor sleep is common among caregivers, but the relationship between sleep and biomarkers of neurodegeneration in this population is unknown. In a secondary analysis of data from a behavioral sleep intervention trial among caregivers of PLWD, we tested the relationship between subjective and objective sleep and plasma biomarkers of neurodegeneration and explored the preliminary effects of the intervention on these biomarkers. Participants included 18 caregivers of PLWD who received a sleep education program (active treatment versus control). Sleep measures included the Pittsburgh Sleep Quality Index, total nights of good sleep (sleep diary), and wrist- actigraphy-measured sleep duration, sleep efficiency (SE), and total wake time (TWT). Plasma biomarkers included amyloid beta-40 (Aβ40), amyloid beta-42 (Aβ42), Glial Fibrillary Acidic Protein (GFAP), Neurofilament light (NfL), and Aβ42/40 ratio. All measures were collected at baseline (n=18), post-intervention (n=11), and three months (n=7). Pearson correlations and t-tests were performed. At baseline, greater (worse) TWT was associated with higher Aβ40 (r=0.60, p=0.009) and Aβ42 (r=0.62, p=0.006) levels. Lower (worse) SE was associated with higher Aβ40 (r= -0.52, p=0.026), Aβ42 (r= -0.61, p=0.008), and NfL (r= -0.48, p=0.043). Less nights of good sleep were associated with a lower Aβ42/40 ratio (r=0.51, p=0.032). Biomarker levels were not significantly different between treatment and control at follow-ups. In conclusion, poor sleep is associated with increased biomarkers of neurodegeneration among caregivers of PLWD. Larger studies are needed to test whether sleep interventions reduce this population’s neurodegeneration risk.

